# Forming a joint dialogue among faith healers, traditional healers and formal health workers in mental health in a Kenyan setting: towards common grounds

**DOI:** 10.1186/s13002-015-0075-6

**Published:** 2016-01-07

**Authors:** Christine W. Musyimi, Victoria N. Mutiso, Erick S. Nandoya, David M. Ndetei

**Affiliations:** University of Nairobi and Founding Director Africa Mental Health Foundation, Mawensi Road, Off Elgon road, Mawensi Garden, Nairobi, Kenya; University of Nairobi, Nairobi, Kenya

**Keywords:** Dialogue, Collaboration, Qualitative, Traditional healers, Faith healers, Clinicians

## Abstract

**Background:**

Qualitative evidence on dialogue formation and collaboration is very scanty in Kenya. This study thus aimed at the formation of dialogue and establishment of collaboration among the informal (faith and traditional healers) and formal health workers (clinicians) in enhancing community–based mental health in rural Kenya.

**Methods:**

Qualitative approach was used to identify barriers and solutions for dialogue formation by conducting nine Focus Group Discussions each consisting of 8–10 participants. Information on age, gender and role in health care setting as well as practitioners’ (henceforth used to mean informal (faith and traditional healers) and formal health workers) perceptions on dialogue was collected to evaluate dialogue formation. Qualitative and quantitative data analysis was performed using thematic content analysis and Statistical Package Social Sciences (SPSS) software respectively.

**Results:**

We identified four dominant themes such as; (i) basic understanding about mental illnesses, (ii) interaction and treatment skills of the respondents to mentally ill persons, (iii) referral gaps and mistrust among the practitioners and (iv) dialogue formation among the practitioners. Although participants were conversant with the definition of mental illness and had interacted with a mentally ill person in their routine practice, they had basic information on the causes and types of mental illness. Traditional and faith healers felt demeaned by the clinicians who disregarded their mode of treatment stereotyping them as “dirty”. After various discussions, majority of practitioners showed interest in collaborating with each other and stated that they had joined the dialogue in order interact with people committed to improving the lives of patients.

**Conclusion:**

Dialogue formation between the formal and the informal health workers is crucial in establishing trust and respect between both practitioners and in improving mental health care in Kenya. This approach could be scaled up among all the registered traditional and faith healers in Kenya.

## Background

The treatment gap for mental disorders in Low and Middle-Income Countries (LMICs) is more than 85 % [1]. Reduced access to mental health care substantially increases patient and family suffering such as poverty, economic loss and unemployment [2]. In rural Kenya, a large number of populations rely on informal health workers who treat disorders similar to those treated in health care facilities [3]. Therefore, treatment resource relocation could decrease the treatment gap of mental disorders [1] given the dearth or even complete absence of mental health specialists and the relative surplus of traditional and faith healer at the community level [4]. Consequently, formal recognition of traditional and faith healers’ role and strategies to build and enhance positive practice may be beneficial in protecting the wellbeing of the poor [3].

The WHO mental health Global Action Programme Intervention Guide (mhGAP-IG) recognizes non-specialists where traditional and faith healers fall, as a potential resource to reduce the treatment gap in low-resource countries [5] while the WHO mental health action plan 2013–2020 recommends inclusion of traditional and faith healers as a resource in achieving the objectives of that plan [6]. The challenge has been on how best to bring THPs on board in a mentally complementary manner.

Both formal (clinicians) and informal (traditional and faith healers) practitioners have been shown to have the willingness to strengthen collaboration, however there are gaps such as lack of collaborative framework in health care [7]. While most collaborative initiatives have focused on gaining healers’ trust rather than involving formal health workers, there are reports to show that formal health workers collaborating with informal health workers are greatly inspired by the healer’s change from mistrust to interest and there is substantive amount of work they are able to do with just little information [8]. Therefore, in order to promote a feasible relationship between the two systems, respectful collaborative relationships at the district level to promote an equitable collaboration in the interests of improved patient care is necessary [9]. It is crucial to build trust by first making initial contact with the informal practitioners, organizing events to present the objectives of the collaborative initiative, plan programmes and reach conclusions together with participating all formal and informal practitioners [8].

In Africa, most traditional and faith healers are willing to learn and refer patients to health care settings but this is not reciprocated [10, 11]. As a result, healers develop resentment towards the clinicians since they feel unappreciated [7 , 9]. For instance, a study conducted in South Africa on nurses’ attitudes towards traditional and faith-based healing confirmed that, referral from clinicians was based on a patient’s interest without discussing the benefits of treatment and thus disregarded THPs treatment [12]. Therefore, referral systems between formal and informal settings are weak and there is need to strengthen them. This collaboration can create complementary systems that are of greater benefit to patients and communities [11].

Qualitative evidence on dialogue formation and collaboration is very scanty in Kenya. Indeed, this is the first study to initiate and subsequently form a joint dialogue consisting of traditional healers, faith healers and clinicians in Kenya. As a result, this study aimed at bridging this gap through identification of barriers and solutions in order to strengthen referral systems and improve patient care at the community level.

Although barriers between formal and informal health systems exist, positive attitude, respect, understanding as well as training using the mhGAP-IG is possible in order for THPs to have basic knowledge on identification and management of priority mental illnesses [13 ]. In addition, all practitioners should endeavor to share common goals of providing patient-centered care and promote the development of protocols to be shared inpatient care [14]. This study formed the baseline phase of a larger project that aimed at training traditional and faith healers on identification and management of depression as a priority condition listed under the mhGAP-IG.

## Methods

This study was conducted in four randomly selected regions in Makueni County, located in the arid and semi-arid areas of Eastern Kenya. It utilized both qualitative and quantitative data collection techniques.

Focus group discussion questions and the guidelines on how to conduct the discussions were developed using the context-specific meaning-making concept [15] by a team of community mental health researchers including a psychiatrist, psychologist, nurse, individuals with a background in community development and a lay person. They included a probing checklist of a summary of topics listed in Table 1. However, new questions were appropriately added to ensure clarification among the participants. All Focus group discussions (FGDs) were conducted indoors to ensure privacy and anonymity; and were led by a researcher, fluent in the local language and experience in conducting qualitative research and community mental health.

**Table 1:** Probing checklist of a summary of topics included in the Focus Group Discussions (FGDs)

Number	Topic
1.	Definition and causes of mental illness with emphasis on depression (a priority mental disorder)
2.	Practitioners’ encounter with patients suffering from mental illness in their routine practice and in their communities
3.	Barriers and solutions encountered during interaction with the different practitioners and care of their patients
4.	Practitioners’ views on a joined seminar/workshop to enhance collaboration

Focus group discussion questions and guides, evaluation of dialogue questionnaire and consent forms were translated into the local language of the participants “*Kikamba*” and then back translated into English by an independent linguist in order to rule out any word inconsistencies between the two multi-lingual speakers.

### Ethics, consent and permissions

Before each FGD and administration of questionnaires, all the participants were explained to the procedures, consequences associated with the study and given the free will to participate in the study. All participants who agreed to participate provided written consent. There were no refusals recorded. All FGDs were audio-taped and involved scribes who were actively involved in note-taking in order to capture all information that cannot be translated into text using audio tapes.

To facilitate dialogue formation, eight FGDs each consisting of 8–10 participants and lasting between forty minutes to one hour were conducted in two phases (summarized in Table 2). The first phase involved independent discussions with each group of traditional healers, faith healers and clinicians (registered nurses and clinical officers with a diploma in nursing and clinical medicine respectively) about their perceptions on mental illness, causes and management with emphasis on depression and the need for collaboration. The separate discussions that consisted of two FGDs per group were held to identify barriers within each group of practitioners. In order to achieve a variation of diverse opinions, a minimum of eight different traditional healers with a male to female ratio of 1:1 were selected for each set of the two FGDs using simple random sampling by shuffling of numbered papers from a list of 50 available and willing healers provided by the region representative. The same procedure was also performed for faith healers. A minimum of two clinicians were selected from each facility including the in-charge and at least one off-duty clinician, to ensure the clinic was still running during the discussions.

**Table 2:** Outline of practitioners’ FGDs

Phase	Type of practitioner	Number of FGD/Discussions	Aim
Phase one	Traditional healers	Two	To discuss practitioners’ perceptions on mental illness, causes and management
	Faith healers	Two	
	Clinicians	Two	
Phase two	Traditional healers and clinicians	One	To reduce mistrust, enhance respect and address any barriers inherent during referrals between the formal and the informal sectors
	Faith healers and clinicians	One	
Evaluation of dialogue formation phase	Traditional healers, faith healers and clinicians	Evaluation of dialogue formation discussions	To evaluate the process of dialogue formation

In phase two, separate FGDs (two) were conducted. The first FGD consisted of 5 traditional healers and 4 clinicians while the second consisted of 5 faith healers and 4 clinicians. This was crucial in order to reduce or alleviate mistrust, enhance respect and address any barriers inherent during referrals between the formal and the informal sectors. The number of FGDs was predetermined at the beginning, however the saturation was nearly reached at the end of phase one especially during the second set of FGDs when barriers and solutions were similar across the practitioners.

In a separate forum, evaluation of dialogue formation was performed among 30 randomly selected participants from each set of practitioners (9 clinicians, 9 faith healers and 12 traditional healers) using a questionnaire containing questions on ensuring community dialogue or collaboration. This questionnaire was adapted from Community Relations Service department of Justice Component in the United States that helps resolve conflicts within the community through non-coercive and third party intervention [16]. Information on age, gender and role in health care setting as well as their perceptions on dialogue was collected. The ratio of male to female was 1:1 and the age was evenly distributed (mean = 43.52 years; range 27–65 years; *SD* =10.45).

This study protocol was approved by the Kenyatta National Hospital and University of Nairobi ethics review committee.

## Data analysis

### Qualitative and quantitative data analysis

The audio-taped FGDs were transcribed verbatim, translated back into English and again into “*kikamba”* in order to retain the original meaning. Thematic content analysis that involved line by line coding by two independent researchers was used. Open coding without any predetermined information on the codes was used but excluded any deviations from the topic of discussion. All emerging themes were selected by a team of researchers, similar categories grouped together and any duplicates crossed out.

Data on evaluation of dialogue was both qualitative and quantitative. It was double-entered by two independent data entry clerks into SPSS for windows version 21.0, compared for inconsistencies in excel and errors corrected. Descriptive statistics were performed using SPSS for windows version 21.0.

## Results

From the thematic content analysis the following four themes were identified; (1) Basic understanding about mental illnesses; (2) Interaction and treatment skills of the respondents to mentally ill persons; (3) Referral gaps and mistrust among the practitioners; (4) Dialogue formation among the practitioners.

### Basic understanding about mental illness by the respondents

Most of the respondents (traditional healers, faith healers and clinicians) had basic information on definition, causes and types of mental illness. The respondents attributed mental illness to substance abuse, physical illnesses, injury and old age. However, attribution to witchcraft was pronounced among traditional and faith healers while poor upbringing was mentioned by the clinicians. The definition and types of mental illness were brought out clearly by the respondents as follows;

**Clinician:***Mental illness is a condition that distorts the normal brain function of an individual or any deviation from the normal interaction of people in the society. There are numerous causes of mental illness such as dependency on drugs, one’s environment, life stressors and poor upbringing.*

**Clinician:***Anxiety, psychosis and schizophrenia are mental disorders. We have behavioral disorders for instance the pedophilias, sadists and great manias. These are actually deviations of individual from the norms of the society.*

Traditional and faith healers similarly defined and classified mental illness as follows;

**Traditional healer:***Mental illness is when an individual behaves abnormally. This might have been inborn or acquired as one is growing up or through witchcraft. It can also be as a result of substance abuse like alcohol, bhang (cannabis) and Miraa (khat) or other diseases such as cerebral malaria, syphilis, diabetes or through high blood pressure that affect the brain. Examples include epilepsy, drooling, unexplained silence, talking aimlessly or hitting people and madness.*

**Faith healer:***Mental illness is a state of mind that makes an individual to act abnormally. It can be caused by witchcraft, demonic powers, drug abuse, disability from birth, old age or head injury causing brain damage. The types include depression, loss of memory, confusion, demonic, running and talking aimlessly, silence and staring blankly.*

### Interaction and treatment skills of the respondents to mentally ill persons

All the respondents had an interaction with a mentally ill person in their routine practice and in their communities. Most respondents in all the FGDs indicated to have developed their skills through supporting and caring for the mentally ill persons. Below is a dialogue indicating the interaction of the respondents with a mentally ill person.

**Facilitator:***In other words you have encountered them in the family level, community level and workstations.*

All practitioners fervidly said in unison *“Yes”*

**Facilitator:***Do you come across people with depression or low mood in the course of your work?*

**Faith healers:***(in unison) yes*

**Faith healer:***We also meet people who are depressed because they have sick people in their families and others are depressed because they are sick.*

### Referral gaps and mistrust among the practitioners

One of the traditional healers felt that they see a majority of patients and that clinicians had little role to play in the care of patients. Generally, traditional healers were willing to work with clinicians although they felt that clinicians neglected and chased away their patients on referral.

**Traditional healer:***We can have a meeting and discuss these issues we encounter with them (i.e. clinicians) but they despise us, view us as useless people and are too proud to meet with us yet traditional medicine has cured many people. For instance, if you can carry out a survey, you will find that in areas where there are many traditional healers, the hospitals around there do not have many patients admitted in their wards. But in areas with no traditional healers, the hospitals are full of patients admitted in wards.*

**Traditional healer:***We have never interacted with the clinicians, they discriminate us saying we are witches and that we are dirty. If you refer a patient to them, they send them away saying they have taken herbs. They abuse us and refuse to work with us.*

**Traditional healer:***They tease the patients we refer to them by discrediting their use of herbs and warning the patients they might die if they continue using herbal medicine. The patient then develops fear and doesn’t come back to us for treatment. The same clinicians come to us when they are sick and having used the medicine at the hospital and their situation hasn’t changed or has worsened, we give them our medicine and they get cured.*

We found that most traditional healers comfortably refer among themselves as quoted by a traditional healer below;

**“***We as traditional healers do not have problems with each other, we work harmoniously. When I realize that the problem with the patient is beyond me, I refer them to another traditional healer whom I know will solve their problem and vice versa. Some clients are also referred to me by other traditional healers and this is how we work.”*

Faith healers felt that there was a gap in the roles played by each practitioner and that some patients literally failed to visit the health centers even on referral and sometimes they end up using the administration to facilitate patients’ referral. Others felt that referring patients for treatment would conflict with their religious belief. However, they acknowledge that awareness and clear-cut roles of each practitioner is imperative. The statements below by faith healers affirm the gap existing in this sector;

*“If a patient believes their sickness is due to demons and they refuse to go to hospital even when we refer them, we sometimes inform the chief or the community health workers to go and get them and take them to the hospital. There are also some religions that do not take the sick to hospital, they believe that sicknesses should be healed by God and if they cannot be healed then they just stay with them.”*

*“Awareness should be created among people on what issues should be addressed by the faith healers, traditional healers and clinicians.”*

*“The clinicians should also be told what our role is to avoid friction with them. They sometimes think we interfere with their work.”*

Clinicians on the contrary seldom refer patients to traditional or faith healers since their patients would demean them and there are no structures in place that allow cross referral. Although there seems to be a gap, they are amenable to structure change. The opinions below were aired out by different clinicians regarding their role in referring patients to traditional or faith healers.

**“***We rarely refer patients to faith healers or traditional healers. The patients can choose to go, but we do not initiate the referral conversation”*

**“***I have heard traditional and faith healers referring patients to the health facility but not the other way since the health care system does not allow.”*

Another clinician pointed out that**, “***sometimes we feel that the patient would not have confidence in us if we referred patients to traditional or faith healers.”*

Two clinicians acknowledged that there was a gap in the chain of referral.

**Clinician**: A*ccording to the chain of referral in the Ministry of Health there is no statement about referring a patient to a faith or traditional healer.*

**Clinician**: *There has never been a common consensus on what role each entity plays, especially on the entity of the traditional and faith healers. It is not clear how a patient benefits from them or how we can be able to relate. We know they play a role in their own aspect but our goal is to make sure the clients reach access to services. So we need to establish structures so that we to know how to relate in linking up the patient to access health care.*

From the discussions, traditional healers stated that they do not express any resentment towards clinicians. On the other hand, the clinicians hate them, regard their treatment as dirty and believe that they have an ulterior motive towards their patients. Two emotive traditional healers said;

“*There is no exchange of referral letters. There is no hospital that gives referral letters to take to a traditional healer. They hate us and cannot accept to give referral letters.”*

“*We love the clinicians but they hate us because they say we are dirty because of our mode of treatment.”*

One traditional healer noted that pastors and clinicians should approach issues without pride.

*"Clinicians are proud because they say they are learned and they despise us. The approach should be that of servant leadership, it is the community we are serving."*

### Dialogue formation among the practitioners

There were no referrals inherent from traditional healers, faith healers to clinicians and vice versa. However, majority of these groups showed interest in collaborating in order to provide effective services. One traditional healer said that it would be very helpful in order to enhance their working relationship for the benefit of the patient and also develop mutual respect for each other instead of clinicians looking down upon them. The willingness to work together among all practitioners was very clear in the joint dialogue.

A nurse in charge of one of the remote centers in the study sites passionately stated that, *"The success that may also come from this discussion and modalities of working is that; addressing the needs of the client will be hastened, and there will be a structured referral system of the client hence reducing delay in service provision. There will also be clear roles for each group to avoid overlap. The faith healer should not dictate that he talks to the God and the clinician to acknowledge that spiritual people have some role to play among their patients because we are living in a diverse society."*

***A faith healer said, “****It is very important that we meet. There is a need for the clinicians to offer their services in churches when called upon by the faith healers and also to visit the community in households to give health care talks. This joint meeting will help us understand each other’s’ role better and how best we can work together.”*

The clinicians emphasized that it is imperative for traditional and faith healers to refer patients to the clinic especially in cases of severe mental illnesses and felt the need to work together. The faith healers pointed out that there are times when the cases need medical attention and thus referral is warranted.

Although it was still difficult for the clinicians to refer patients to the traditional and faith healers due to lack of structures, they acknowledged that a dialogue was crucial in order to bring together all the stakeholders in mental health and work collaboratively.

The following are statements from each practitioner in regards to collaboration;

***Clinician:****I would say that traditional and faith healers are a resource within the community and can address the issues of a patient wellbeing. So I think they can be brought on board to have conformity.*

***Traditional healer:****The patients will benefit because they will not lack treatment since we will work together to solve any difficult issue and refer when necessary. Everyone will also be exercising their specialization for the benefit of the patient.*

***Another traditional healer said,****“We should involve all stakeholders in such an approach, especially in the training and education sessions. We should involve people like the doctors from hospitals, faith healers, and traditional healers.”*

***Faith healer:****Unless we understand each other and respect each other’s work, there are bound to be problems. These problems can only be solved if we sit together and sort out the issues and a working program developed to avoid friction. Having joint meetings will reduce problems since we will talk and solve the issues affecting us.*

### Evaluation of dialogue formation

All the 30 participants (100 %) stated that they joined the dialogue in order to come together with a group of people committed to improving the lives of patients and not because it was a way of spending their free time or they were not busy at their workplace. This is an indication that health care providers (formal and informal practitioners) have a common goal and are ready to work collaboratively to improve the lives of patients.

The health care providers had various expectations for the dialogue with some having multiple expectations. They included; to add new knowledge (23 %), to interact with other health care providers (43 %) and to improve their work with respect to patient care (47 %). Out of the 30 participants, 26 (96 %) of the participants’ expectations were satisfactorily met.

In addition, most health care providers added that they were comfortable participating in the discussion, the dialogue climate was positive and respectful and it gave them new insights about how to improve patient care. They also found the dialogue to be a valuable experience and would like to participate in a future session as the experience motivated them to act differently. Respect of other people’s opinion was seen as a very salient component for dialogue meetings.

## Discussion

Basic understanding of the traditional healers, faith healers and clinicians about mental illness, interaction of health care providers with mentally ill persons and identification of referral gaps and mistrust among the practitioners were key aspects which this study delved into in order to establish a basis for dialogue and collaboration among the practitioners in provision of mental health services in the local community context. Dialogue formation was a process and required identification of barriers at the initial stage that guide the formation of joint dialogue. It is therefore imperative to look at requirements necessary for collaboration in order to enhance sustainability [7].

Of worthy to note was the interaction of the practitioners with mentally ill persons both in the community and during their care of patients. Studies conducted in informal settlements in Kenya have shown that traditional and faith healers see a high number of patients with priority mental disorders such as depression, anxiety and psychosis [17, 18]. Similarly, clinicians encounter such patients in their routine primary health care [19, 20]. This is thus an opportunity to provide training using evidence-based practice to such groups in order to reduce the huge mental health treatment gap.

We found that conducting discussions with independent groups of practitioners identified gaps necessary for collaboration. Most practitioners acknowledged that there were referral, treatment and training gaps and therefore collaboration could improve patient care since they all had similar goals towards their patients and they were both willing to learn from each other. Earlier literature has found that collaboration of traditional healers with clinicians improves knowledge, skills, confidence and transparency of their work in their communities and timely referral of their patients [21].

There was unidirectional referral of traditional and faith healer patients to clinicians and this made the practitioners unappreciated and demeaned. This was consistent with findings from South Africa on collaboration between traditional and biomedical health care systems [11]. However effective collaboration between the two sectors, a technique that was employed in this study can strengthen the referral systems [11].

It was noted that training was crucial in order to bridge the gap and therefore all stakeholders warranted training to enhance collaboration. Mistrust among practitioners and patients hinder the development of effective partnerships [7, 22]. Majority of the practitioners showed concerted effort to promote collaboration by emphasizing on the need for referral system and importance of establishing the roles of the different practitioners in health care. Various studies have been demonstrated in Africa to show the willingness of traditional and faith healers in collaborating with clinicians [11, 22–25]. Kaboru and colleagues stated that education and adequate community involvement could enhance this collaboration [7]. In addition, formal agreements are necessary in order to protect intellectual rights and accountability as well as the promotion of cultural awareness among the mental health practitioners [24]. This will consequently, ameliorate access to care, decrease the burden of mental illness among patients, their families and the community [26].

When dialogue was evaluated, health care providers had a common goal and were ready to work collaboratively to improve the lives of their patients. They were also enthusiastic as dialogue gave them new insights and motivated them to act differently. A study conducted in Zambia on practitioners’ experiences and attitudes towards collaboration showed that 40 % of Biomedical Health Providers (BHP) were willing to work closely with traditional health practitioners [7].

Although barriers between formal and informal health systems exist, positive attitude, respect, understanding and training using the mhGAP-IG is possible in order for traditional and faith healers to understand identification and management of priority mental illnesses [13]. Burns and Tomito in South Africa have recommended the inclusion of innovative programmes as strategies that foster collaboration between the two sectors and improve mental health care in Africa [27].

## Conclusion

There is lack of suffice information on the process of initiating collaboration between the formal and the informal sectors. The step by step process employed in this study is imperative in initiating, enhancing collaboration and reducing the mistrust inherent between the two systems. Through mutual understanding in each phase of dialogue formation, all practitioners were ready to work collaboratively to improve the lives of their patients and community.

The basic understanding of mental illness and the ability of traditional and faith healers to interact with mentally ill patients in their routine practice could be used as an opportunity to promote their training needs as well as licensing them in order to improve transparency and formalize their practice. Their willingness to collaborate with the clinicians is also a potential channel for establishing stable partnerships in order to reduce the mental health treatment gap through establishment of an apt chain of referral.

It is necessary for traditional healers, faith healers and clinicians to continuously engage in respectful dialogue and open two-way dialogue to understand each other and build mutual respect and trust to move forward with medical pluralism and increase mental health outcomes in patients. This approach could be scaled up among all the registered traditional and faith healers in Kenya.

## References

[1] Demyttenaere K, Bruffaerts R, Posada-Villa J, Gasquet I, Kovess V, Lepine JP (2004). Prevalence, severity, and unmet need for treatment of mental disorders in the World Health Organization World Mental Health Surveys. Jama.

[2] Ngui E, Khasakhala L, Ndetei D, Roberts L (2010). Mental disorders, health inequalities and ethics: A global perspective. Int Rev Psychiatry.

[3] Lambert J, Omindi-ogaja E, Gatheru G, Mirangi T, Owara J, Herbst CH, et al. The Contribution of Traditional Herbal Medicine Practitioners to Kenyan Health Care Delivery Results from Community Health-Seeking Behavior Vignettes and a Traditional Herbal Medicine Practitioner Survey [Internet]. World Bank, Washington, DC. © World Bank. 2011 [cited 2016 Jan 5]. Available from: https://openknowledge.worldbank.org/handle/10986/13588 License: CC BY 3.0 Unported

[4] Ndetei D, Ongetcha F, Mutiso V, Kuria M, Khasakhala L, Kokonya D (2007). The challenges of human resources in mental health in Kenya. Afr J Psychiatry.

[5] World Health Organization. Mental Health Gap Action Programme: mhGAP Intervention Guide for Mental, Neurological and Substance Use Disorders in Non-specialized Health Settings. 1.0 ed. Geneva: World Health Organization; 2010. p. 1–65. 23741783

[6] World Health Organization. Mental health action plan 2013–2020 [Internet]. 2013. p. 1–48. Retrieved July 22, 2015, from http://apps.who.int/iris/bitstream/10665/89966/1/9789241506021_eng.pdf.

[7] Kaboru BB, Falkenberg T, Ndulo J, Muchimba M, Solo K, Faxelid E (2006). Communities’ views on prerequisites for collaboration between modern and traditional health sectors in relation to STI/HIV/AIDS care in Zambia. Health Policy.

[8] Joint United Nations Programme on HIV/AIDS (UNAIDS). Collaborating with Traditional Healers for HIV Prevention and Care in sub-Saharan Africa: suggestions for Programme Managers and Field Workers [Internet]. UNAIDS best practice collection. Geneva: World Health Organization; 2006 [cited 2015 Jan 6]. Available from: http://data.unaids.org/publications/IRC-pub07/jc967-tradhealers_en.pdf

[9] Campbell-Hall V, Petersen I, Bhana A, Mjadu S, Hosegood V, Flisher A (2010). Collaboration between traditional practitioners and primary health care staff in South Africa_ developing a workable partnership for community ment. Transcult Psychiatry.

[10] Green EC (2000). Traditional Healers and AIDS in Uganda. J Altern Complement Med.

[11] Mngqundaniso N, Peltzer K (2008). Traditional Healers and Nurses_ A Qualitative Study on Their Role on Sexually Transmitted Infections Including HIV and AIDS in KwaZulu-Natal, South Africa. Afr J Tradit Complement Altern Med.

[12] Peltzer K, Khoza L (2002). Attitudes and knowledge of nurse practitioners towards traditional healing, faith healing and complementary medicine in the Northern Province of South Africa. South African J Nurs.

[13] Uwakwe R, Otakpor A. Public Mental Health - Using the Mental Health Gap Action Program to Put all Hands to the Pumps. Front Public Heal. 2014;2(33). Available from: http://www.ncbi.nlm.nih.gov/pubmed/?term=Public+Mental+Health+−+Using+the+Mental+Health+Gap+Action+Program+to+Put+all+Hands+to+the+Pumps. 10.3389/fpubh.2014.00033 PMC4000990 24795874

[14] Chung V, Ma P, Lau C, Griffiths S (2012). Developing policy for integrating biomedicine and traditional chinese medical practice using focus groups and the delphi technique. Evid Based Complement Altern Med.

[15] Wilkinson S, Smith JA (2003). A practical guide to research methods. Qualitative psychology.

[16] U.S. Dept. of Justice, The Community Relations Service. Community Dialogue Guide: Conducting a Discussion on Race [Internet]. 2003 [cited 2015 Jul 22]. Available from: https://www.ncjrs.gov/App/publications/abstract.aspx?ID=202941

[17] Ndetei D, Khasakhala L, Kingori J, Oginga A, Raja S. The complementary role of traditional and faith healers and potential liaisons with western-style mental health services in Kenya [Internet]. 2008 [cited 2015 Apr 28]. p. 1–22. Available from: https://www.uonbi.ac.ke/ndetei/files/the_complementary_role_of_traditional_and_faith_healers_and_potential_liaisons_with_western-style_mental_health_ser

[18] Mbwayo A, Ndetei D, Mutiso V, Khasakhala L (2013). Traditional healers and provision of mental health services in cosmopolitan informal settlements in. Afr J Psychiatry.

[19] 19.Ndetei D, Khasakhala L, Kuria M, Mutiso V, Ongecha-Owuor F, Kokonya D. The prevalence of mental disorders in adults in different level general medical facilities in Kenya: a cross-sectional study. Ann Gen Psychiatry. 2009;8(1). Available from: http://www.ncbi.nlm.nih.gov/pmc/articles/PMC2631009/10.1186/1744-859X-8-1 PMC2631009 19144164

[20] Jenkins R, Njenga F, Okonji M, Kigamwa P, Baraza M, Ayuyo J (2012). Prevalence of common mental disorders in a rural district of Kenya, and socio-demographic risk factors. Int J Environ Res Public Health.

[21] King R. Collaboration with traditional healers in HIV/AIDS prevention and care in sub-Saharan Africa: A literature review [Internet]. Joint United Nations Programme on HIV/AIDS (UNAIDS) Best Practice Collection. Geneva: World Health Organization; 2000 [cited 2015 Jan 6]. Available from: http://www.hst.org.za/uploads/files/Collab_Lit_Rev.pdf.

[22] Audet CM, Hamilton E, Hughart L, Salato J (2015). Engagement of Traditional Healers and Birth Attendants as a Controversial Proposal to Extend the HIV Health Workforce. Curr HIV/AIDS Rep.

[23] Birhan W, Giday M, Teklehaymanot T (2011). The contribution of traditional healers’ clinics to public health care system in Addis Ababa, Ethiopia: a cross-sectional study. J Ethnobiol Ethnomed.

[24] Keikelame MJ, Swartz L. “A thing full of stories”: Traditional healers’ explanations of epilepsy and perspectives on collaboration with biomedical health care in Cape Town. Transcult Psychiatry [Internet]. 2015 Mar 13; Available from: http://www.ncbi.nlm.nih.gov/pubmed/25680366 10.1177/1363461515571626 PMC4552613 25680366

[25] Mohamed-Kaloo Z, Laher S (2014). Perceptions of mental illness among Muslim general practitioners in South Africa. S Afr Med J.

[26] Schoonover J, Lipkin S, Javid M, Rosen A, Solanki M, Shah S (2014). Perceptions of Traditional Healing for Mental Illness in Rural Gujarat. Ann Glob Heal.

[27] Burns JK, Tomita A (2015). Traditional and religious healers in the pathway to care for people with mental disorders in Africa: a systematic review and meta-analysis. Soc Psychiatry Psychiatr Epidemiol.

